# Operational Improvement of a Proton Therapy System From Reduced Energy Layer Switching Time

**DOI:** 10.1016/j.ijpt.2025.100742

**Published:** 2025-02-25

**Authors:** Mark E. Artz, Hardev S. Grewal, Yawei Zhang, Perry B. Johnson, Mohammad Saki, Niek Schreuder, Eric D. Brooks, Jiyeon Park

**Affiliations:** a Department of Radiation Oncology, University of Florida College of Medicine, Jacksonville, Florida, USA; b Leo Cancer Care, Middleton, Wisconsin, USA; c Premier Radiation Oncology Associates, Clearwater, Florida, USA

**Keywords:** Proton Therapy, Energy layer switching time, Treatment capacity improvement, Proton beam delivery time

## Abstract

**Purpose:**

This study reports the potential operational improvement provided by reduced energy layer switching time and total beam delivery time of a 3-room IBA ProteusPLUS system.

**Materials and methods:**

Eight treatment fields, including various disease sites and a reference cube, were analyzed for energy layer switching time and total delivery time before and after an energy layer switching time upgrade. The upgraded beam delivery time estimated the total treatment capacity for a 12-h treatment day using a 3-room system with a single common cyclotron. The potential increase in revenue was estimated using Medicare reimbursement rates for proton therapy intermediate.

**Results:**

The average layer switching time was reduced from 6.33 to 1.86 s, reducing the average field delivery time from 240 to 113 s. Overall, the average energy layer switching time reduction was 70.6%, and the average total treatment field delivery time was reduced by 52.6%. Head and neck cancer fields experienced the greatest treatment time reduction of 59.5%, reducing from 236 to 95.5 s. The treatment field delivery time reduction allowed an increase from 60 to 127 patients per day, an increase of 112%.

**Conclusion:**

Reductions in energy layer switching time significantly decreased treatment field delivery time. Reduced treatment field delivery time may provide clinical benefits to patients through improved patient alignment and comfort. Increased treatment capacity could also improve patient access to proton therapy for disease sites that may experience clinical benefits, such as head and neck cancer.

## Introduction

Proton therapy machines designed initially for scattered beams and later converted to support pencil beam scanning (PBS) often suffer from much longer treatment field delivery times than systems designed solely for PBS. Early proton therapy systems, particularly those with multiple treatment rooms (3-5), often rely on a single shared cyclotron, leading to beam scheduling conflicts. For patients requiring multiple fields per treatment with each field taking 2 to 5 min to deliver, a single treatment session may occupy up to 15 min of beam time. This limits beam availability for other rooms, which are dependent on the same cyclotron. In contrast, newer single-room systems, like the ProteusONE (Ion Beam Applications, Louvain-la-Neuve, Belgium) and S250-FIT (Mevion Medical Systems, Littleton, MA), have dedicated accelerators for each room thereby eliminating such conflicts.

Eddy currents are induced when steel-cored electromagnets experience a change in magnetic field; the energy layer switching time depends on how quickly these eddy currents decay. The IBA Proteus PLUS treatment machine at the University of Florida Health Proton Therapy Institute (UFHPTI) operates on platform C architecture, which is shared with Seoul in South Korea, a later revision on the IBA ProteusPLUS system in operation at Mass General Hospital. This system was adapted to PBS from passive scattered beams. More recent Proteus Plus systems, such as those built in Knoxville and Miami, have beamline dipole magnets designed with thinner laminations in the steel beamline dipole magnets to reduce induced eddy currents when ramping the electromagnets between energy layers. Since the UFHPTI dipole magnets were initially designed for double scattering, which did not require energy layer switching, they have thicker laminations in the steel of the beamline dipole magnets requiring more time to regulate and stabilize the dynamic magnetic field due to the eddy current effect to decay after ramping down the excitation current when switching between energy layers.

This study outlines several improvements to the UFHPTI ProteusPLUS system to enhance its operational efficiency. These include changes to the scanning magnet controller and dipole magnet power supplies. The most significant change was a reduction in the dipole magnet settling time when changing between energy layers. To utilize the same magnet hysteresis, the beamline is ramped down from the highest to the lowest energy setting. This results in the most significant energy change from the system’s maximum energy down to the energy required for the deepest layer in the treatment field. In contrast, each layer change within the field demands less adjustment in magnitude. The total field delivery time could be significantly reduced by reducing the time between energy layers to allow magnet settling from small energy changes only.

This study aims to evaluate the impact of reducing energy layer switching time on overall treatment efficiency, patient capacity, and revenue generation in a multiroom proton therapy system. Such improvements could address limitations commonly observed in older proton therapy systems initially designed for passive scattering and later adapted for PBS, there are approximately 10 of these such systems in the United States and several others worldwide. The concepts around treatment capacity limitations caused by shared beams and extended field delivery times discussed in this work are also relevant to 2-room proton therapy systems, multiroom synchrotron-based systems, and even modern single-room systems may benefit from reductions in energy layer switching time for treatment fields with >30 energy layers.

## Materials and methods

PBS treatment fields were created to match various disease sites, including a reference dosimetry field to deliver 2 Gy by the IAEA protocol^1^ (Ref Cube), breast, prostate with lymph nodes (P+LN), and head and neck cancer (H&N). These fields were delivered before and after implementing the technical advancements to improve the layer switching time using site configuration 6.5.1 before and 6.5.3 after.

System beam delivery logs were analyzed for each field delivery, providing the energy layer switching time with and without tuning. The layer switching time was reported with tuning time included for beam delivery data analyzed from version 6.5.3 as it most closely resembles the layer switching time calculated from beams previously delivered with version 6.5.1.

Due to limitations in version 6.5.1 system logs, energy layer switching times were estimated using recorded audio generated during the pulse counting process at the start and end of each layer of the Dosimetry counter electronic unit, with analysis in a script written in MATLAB (MathWorks Inc, Natick, MA) to determine layer switching durations. This approach provided a practical solution for estimating switching times without detailed logs.

To estimate the impact of treatment capacity, a 3-room proton system was assumed with a single shared cyclotron, similar to the site configuration at UFHPTI. Each patient was taken to receive treatment through 3 fields, a conservative estimate for the required time per patient since the beam was modeled as continuously in use. This configuration enables 1 treatment beam to be administered 1 room at a time. However, the other rooms are ready to treat patients once alignment with x-rays is completed. Since patient alignment can be completed in parallel across treatment rooms, only the fundamental underlying limit of the series beam delivery was used to calculate the treatment capacity limit of a 3-room system, that is, the total beam delivery time required to treat patients in the other 2 rooms is sufficient to complete patient setup in the third room.

Medicare reimbursement rates for proton therapy intermediate, code 77523,^2^ were used to model the potential revenue for a 3-room proton therapy system operating at total capacity for an entire 12-h day. Although private insurance reimbursement can vary greatly, the Medicare reimbursement approach provides a conservative estimate. It serves as a foundation for assessing potential revenue, given that Medicare data are publicly available and often forms the baseline for reimbursement policies. The reimbursement rate used for this study was $1351 per fraction.^2^

Internal rate of return (IRR) is a commonly used metric in project evaluation that iteratively calculates the discount rate that would produce a net present value of 0.^3^ IRR is a simple way to compare projects in terms of equivalent compound interest from other investment opportunities such as stocks or bonds. The rate of return available from other projects and investments often informs the hurdle rate, the minimum rate at which projects are pursued. Hurdle rates >10% to 20% are often used for selection of projects based on IRR. IRR was calculated using a total project cost estimate of $120 million based on the average bond financing across 3 proton therapy projects with similar 3-room configurations.^4^ The cost per room, approximately $40 million, also aligns well with recent single-room proton system project costs.^5^ The cost was assumed to be spread evenly over 3 years with a 10% operating margin in subsequent years.

## Results

**Figure 1:**
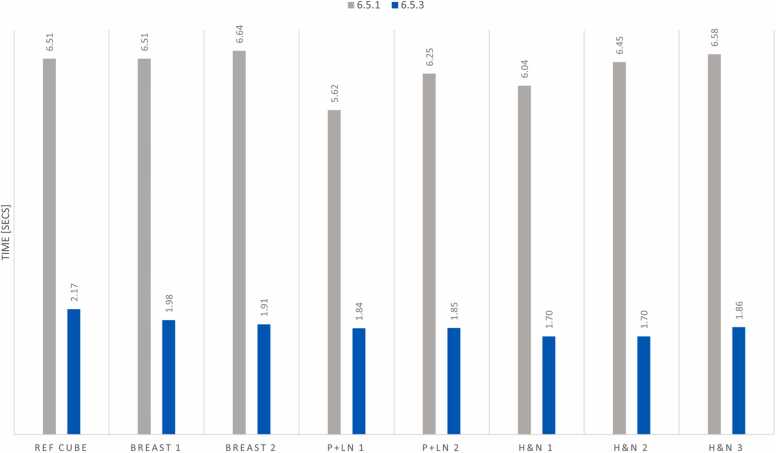
The averaged energy layer switching time of treatment fields before and after the upgrade. Head and neck fields demonstrated the most significant improvement in averaged energy layer switching time, 6.58 s reduced to 1.70 s.

**Figure 2:**
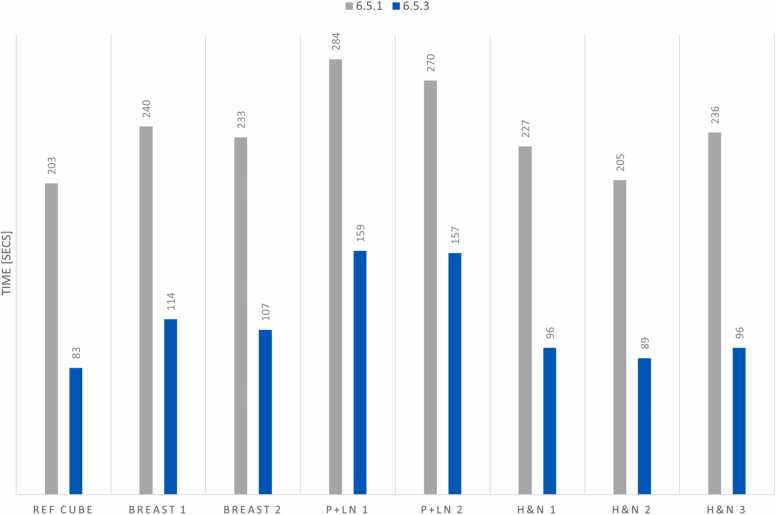
Displays total treatment field delivery time before and after the upgrade. Head and neck fields demonstrated the most significant reduction in total treatment delivery time, with 236 s reduced to 96 s.

Figure 1 presents each test treatment field's average layer switching time before and after the system upgrade (site configurations 6.5.1 [original] and 6.5.3 [updated]). Additionally, the total treatment delivery time for each of the Ref Cube, Breast, P+LN, and H&N sites is shown in Figure 2. Among these sites, H&N treatment fields experienced the most significant overall reduction in delivery time, achieving up to a 59.5% decrease. P+LN fields showed the most minor improvement in beam delivery time at 44.1%, summarized in Table 1.

**Table 1:** The total delivery time and energy layer switching time of treatment fields are shown with the percent difference.

Field type	Version	Beam	Field total (s)	Difference (%)	Layer average (s)	Difference (%)	Field information
Ref cube							
	6.5.1	1A1	203.0		6.51		− 20 layers − 171.9-115.1 MeV
	6.5.3	1A1	82.6	−59.3	2.17	−66.7	
Breast							
	6.5.1	1A1	240.0		6.51		− 30 layers − 178.2-104.8 MeV
	6.5.3	1A1	114.2	−52.4	1.98	−69.6	
	6.5.1	1B1	233.0		6.64		− 30 layers − 178.2-104.8 MeV
	6.5.3	1B1	107.3	−53.9	1.91	−71.3	
P+LN							
	6.5.1	1A1	284.0		5.62		− 39 layers − 217.9-102.4 MeV
	6.5.3	1A1	158.8	−44.1	1.84	−67.2	
	6.5.1	1B1	270.0		6.25		− 38 layers − 215.9-102.4 MeV
	6.5.3	1B1	157.4	−41.7	1.85	−70.5	
H&N							
	6.5.1	1A1	227.0		6.04		− 35 layers − 168.8-102.4 MeV
	6.5.3	1A1	95.5	−57.9	1.70	−71.9	
	6.5.1	1B1	205.0		6.45		− 35 layers − 158.1-102.4 MeV
	6.5.3	1B1	88.8	−56.7	1.75	−72.9	
	6.5.1	1C1	236.0		6.58		− 37 layers − 175-102.4 MeV
	6.5.3	1C1	95.5	−59.5	1.70	−74.2	
Average							
	6.5.1	All	237.3		6.33		
	6.5.3	All	112.5	−52.6	1.86	−70.6	

**Abbreviations: P+LN, prostate with lymph nodes; H&N, head and neck.**

The average treatment field delivery time was reduced by 124.7 s, and the average energy layer switching time was reduced by 4.5 s.

The average layer switching time was reduced from 6.33 to 1.86 s, reducing average field delivery times from 240 to 113 s. Overall, the energy layer switching time reduction averaged 70.6% and decreased total treatment field delivery time by 52.6%.

Under the previously described estimation conditions, each patient required approximately 12 min per treatment field before the upgrade and 5.65 min after. Completing 1 fractional treatment using 3 fields for a patient in each of the 3 proton treatment rooms connected to the shared cyclotron would require 36.00 min before and 16.95 min after the upgrade. During a 12-h treatment day, this improvement would increase the capacity of the 3-room proton system from 60 to 127 patients per day. This represents a 112% increase in patient throughput after reducing the energy layer switching time.

Using public Medicare reimbursement data for proton therapy intermediate code 77523, the potential daily revenue for a fully utilized 3-room system increased from $81,060 to $171,577 and the potential annual revenue increased from $20,265,000 to $42,894,250 (Table 2). Although the potential revenue increased significantly, the total projected 15-year IRR for a $120 million proton therapy project remained negative, however, improved from −13% to −6%.

**Table 2:** The total delivery time of 3 treatment fields was used to estimate the total treatment capacity of the 3-room proton therapy system depending on a single cyclotron; the total capacity for a 12-h period increased from 60 to 127 patients per day.

	Version 6.5.1	Version 6.5.3	Units	Difference
Average energy layer switching time	6.33	1.86	Seconds	−70.6%
Time per field	240	113	Seconds	−52.6%
Time 3-fields	12	5.65	Minutes	−52.6%
Minimum time for 3-patients	36	16.95	Minutes	−52.6%
Patients in 12-h day	60	127	Patients	+112%
Total treatment charges per day	$81,060	$171,577	USD	+$90,517
Total treatment charges per year	$20,265,000	$42,894,250	USD	+$22,629,250
15yr IRR of $120M 3-room proton center	−13%	−6%	%	+54%

Using the Medicare reimbursement rate for proton therapy intermediate charge code 77523, the total potential revenue increased from $20,265,000 by $22,774,708 to $43,040,708.

## Discussion

Reducing field delivery time offers clinical benefits beyond operational efficiency, including enhanced patient comfort during the shorter table time and improved confidence in patient alignment during the beam on time. Increasing patient treatment capacity increases access to patient care for many proton therapy centers that serve as the only facility in their region. This is particularly important for H&N cancer patients, who have shown clinical benefit from proton therapy compared to intensity-modulated radiation therapy.^6^ Notably, head and neck fields demonstrated the most significant reduction in beam delivery time following the decrease in layer switching time.

The relative delivery time reduction is consistent with predictions from energy layer switching time improvement studies. However, this study's total treatment field delivery time is closer to 2 min than 4 min, as de Muinck Keizer et al^7^ reported. Uncertainty in patient alignment due to intrafraction motion increases significantly as the patients spend longer on the treatment couch.^8^ While the average energy layer switching time reduced from 6.33 to 1.86 s in this study, many newer systems have energy layer switching times <1 s.^9^, ^10^, ^11^ Although these newer systems experience treatment capacity benefits from shorter field delivery times, it is much less pronounced as many of these systems are single rooms and do not share the proton beam; although energy layer switching time still plays a role in total beam delivery and treatment time, other operational constraints, such as patient setup, become increasingly more significant.

The variability of relative reduction in treatment field delivery time may be due to the variability of beam tuning time with energy. The beam tuning time was included in the layer switching time for both version 6.5.1 and 6.5.3 analyses to ensure equivalence. The proton machine beam centering is calibrated at the highest energy and treatment fields with higher energy may require less tuning to reach the isocenter coordinates than lower energy fields. This may explain the lower relative field delivery time reduction observed in higher energy P+LN fields compared to lower energy fields such as H&N.

Despite the gains in treatment capacity, the IRR calculations for the $120 million proton therapy project remain negative when analyzed over a 15-year timeframe. This demonstrates that achieving a positive IRR within 15 years with the modeled Medicare reimbursement and equipment cost may not be possible despite enhancing the revenue potential. Operational improvements, such as reduced treatment times, could be combined with reductions in proton therapy equipment and installation costs to provide potential for projects with positive IRR in the future. Improvements in treatment capacity through upgrades of existing equipment may help to accommodate some of the expected increase in demand for oncology care, which is expected to grow by 34% and reach $245 billion by 2030.^12^

A limitation of this study is the assumption of continuous utilization of the proton therapy beam to estimate the capacity of a 3-room proton therapy system. In practice, there will be times when none of the rooms actively use the beam, thus reducing the number of patients treated daily. Although many proton therapy centers operate more extended hours, up to 14 to 16 h daily, this is often seasonal and not evenly distributed across all rooms. For consistency, a 12-h day was used in this analysis to account for these fluctuations and provide a conservative estimate of capacity. Another limitation of the study is the number of fields analyzed; during the upgrade to version 6.5.3, the ProteusPlus system had to be reverted to 6.5.1 to acquire the reference data, and after the upgrade, it was no longer possible to deliver fields in version 6.5.1.

## Conclusion

Reduced energy layer switching times have significantly improved the efficiency of the average treatment field delivery time, increasing it by up to 52.6%. The most notable reductions in delivery time were observed in H&N treatment fields. By shortening these delivery times, we estimated a potential increase in patient capacity of 112%, using 3 treatment rooms with a single cyclotron configuration. Reducing table time can enhance patient alignment reliability during and between treatment fields. Additionally, the improved delivery efficiency of the treatment beams presents promising opportunities to expand access to more patients who could benefit from the superior clinical outcomes offered by proton therapy.

## Author Contributions

Mark Artz: Conceptualization, Methodology, Software, Validation, Formal Analysis, Investigation, Resources, Data Curation, Writing- Original Draft, Writing- Review and Editing, Visualization, Supervision, Project Administration. Perry Johnson: Resources, Supervision, Project Administration, Writing- Review and Editing. Hardey Grewal: Validation, Formal Analysis, Writing- Original Draft, Writing- Review and Editing. Yawei Zhang: Validation, Formal Analysis, Writing- Original Draft, Writing- Review and Editing. Mohammad Saki: Writing- Original Draft, Writing- Review and Editing. Niek Schreuder: Conceptualization, Writing- Original Draft, Writing- Review and Editing. Eric Brooks: Conceptualization, Writing- Original Draft, Writing- Review and Editing. Jiyeon Park: Conceptualization, Methodology, Software, Validation, Formal Analysis, Investigation, Resources, Data Curation, Writing- Original Draft, Writing – Review and Editing.

## Declaration of Conflicts of Interest

PBJ is an Associate Editor of IJPT. All other authors report no conflicts of interest.

## Data Sharing Statement

The authors agree to share anonymized data upon reasonable request by researchers.
